# Hypoxic Culture Maintains Cell Growth of the Primary Human Valve Interstitial Cells with Stemness

**DOI:** 10.3390/ijms221910534

**Published:** 2021-09-29

**Authors:** Kaho Kanno, Tomohisa Sakaue, Mika Hamaguchi, Kenji Namiguchi, Daisuke Nanba, Jun Aono, Mie Kurata, Junya Masumoto, Shigeki Higashiyama, Hironori Izutani

**Affiliations:** 1 Department of Cardiovascular and Thoracic Surgery, Ehime University Graduate School of Medicine, Toon, Shitsukawa, Ehime 791-0295, Japan; khpn529@gmail.com (K.K.); kenji.n0520@gmail.com (K.N.); 2 Department of Cell Growth and Tumor Regulation, Proteo-Science Center (PROS), Ehime University, Toon, Shitsukawa, Ehime 791-0295, Japan; shigeki@m.ehime-u.ac.jp; 3 Department of Cardiology, Pulmonology, Hypertension, and Nephrology, Ehime University Graduate School of Medicine, Toon, Shitsukawa, Ehime 791-0295, Japan; mtwkhs.mikant@gmail.com (M.H.); junaono0616@gmail.com (J.A.); 4 Department of Stem Cell Biology, Medical Research Institute, Tokyo Medical and Dental University, Bunkyo-ku, Tokyo 113-8510, Japan; nanbscm@tmd.ac.jp; 5 Department of Pathology, Division of Analytical Pathology, Ehime University Graduate School of Medicine, Toon, Shitsukawa, Ehime 791-0295, Japan; miekrt@m.ehime-u.ac.jp (M.K.); masumoto@m.ehime-u.ac.jp (J.M.); 6 Department of Pathology, Proteo-Science Center (PROS), Toon Shitsukawa, Ehime 791-0295, Japan; 7 Department of Biochemistry and Molecular Genetics, Ehime University Graduate School of Medicine, Toon, Shitsukawa, Ehime 791-0295, Japan; 8 Department of Molecular and Cellular Biology, Research Center, Osaka International Cancer Institute, Chuo-ku, Osaka-shi, Osaka 541-8567, Japan

**Keywords:** aortic valve, interstitial cells, hypoxia, oxidative stress

## Abstract

The characterization of aortic valve interstitial cells (VICs) cultured under optimal conditions is essential for understanding the molecular mechanisms underlying aortic valve stenosis. Here, we propose 2% hypoxia as an optimum VIC culture condition. Leaflets harvested from patients with aortic valve regurgitation were digested using collagenase and VICs were cultured under the 2% hypoxic condition. A significant increase in VIC growth was observed in 2% hypoxia (hypo-VICs), compared to normoxia (normo-VICs). RNA-sequencing revealed that downregulation of oxidative stress-marker genes (such as superoxide dismutase) and upregulation of cell cycle accelerators (such as cyclins) occurred in hypo-VICs. Accumulation of reactive oxygen species was observed in normo-VICs, indicating that low oxygen tension can avoid oxidative stress with cell-cycle arrest. Further mRNA quantifications revealed significant upregulation of several mesenchymal and hematopoietic progenitor markers, including CD34, in hypo-VICs. The stemness of hypo-VICs was confirmed using osteoblast differentiation assays, indicating that hypoxic culture is beneficial for maintaining growth and stemness, as well as for avoiding senescence via oxidative stress. The availability of hypoxic culture was also demonstrated in the molecular screening using proteomics. Therefore, hypoxic culture can be helpful for the identification of therapeutic targets and the evaluation of VIC molecular functions in vitro.

## 1. Introduction

Aortic valves are essential for maintaining the proper systemic hemodynamic by preventing backflow to the left ventricle. However, stenotic valves with calcium depositions are often observed in people over the age of 65 years [1]. Valve leaflet are primarily comprised of endothelial cells, myofibroblasts, and valve interstitial cells (VICs). VICs regulate homeostasis of valve leaflet tissues by providing extracellular matrix that increases the valve’s resistance to mechanical stress. Additionally, VICs contribute to valve calcification via their transformation to osteoblastic cells in the progression of aortic valve stenosis [2,3,4]. The expression of osteoblast-specific marker genes has been detected in calcified tissues in human samples. Nevertheless, only a few studies have investigated the biological characteristics of VIC-derived osteoblastic cell in vitro, and the signaling pathways underlying differentiation to osteoblasts remain poorly understood.

Since human bone marrow-derived mesenchymal stem cells (BMSCs) were discovered as contributors to tissue regeneration in mesenchymal tissues obtained from bone, muscle, and adipose tissues [5], tissue-specific mesenchymal stem cells (MSCs), such as adipose-tissue-derived MSCs, have been identified. The abundant existence of progenitor cells of osteoblasts has been demonstrated in aortic valve leaflets. The authors demonstrated that over 40% of mesenchymal progenitors and osteoprogenitors were contained in the primary VICs, which contribute to calcification in aortic valves [6]. Another study suggested that interstitial Cajal-like cells/telocytes (TCs) expressing markers of hematopoietic and MSCs are present in aortic valves, which might contribute to tissue regeneration or repair [7]. To understand the molecular mechanism underlying osteoblast-dependent calcification in aortic valves, detailed characterizations of multipotent VICs are essential.

Many studies have demonstrated that hypoxic conditions are useful for culturing MSCs in terms of maintaining adequate cell growth and stemness based on the evidence that MSC proliferation occurs in regions of low oxygen tension (1–7%) in bone marrow [8,9]. Tsai et al. [10]. found that hypoxic culture promotes MSC proliferation and maintains mesenchymal cell properties by decreasing senescence [10]. Valorani et al. [11] also reported that cell proliferation, differentiation, and migration in human adipose tissue MSCs were potentiated by hypoxia [11]. Hypoxic conditions are also effective for the growth of human skin fibroblasts while preventing chromosomal aberrations [12]. However, little is known about the molecular and cellular characterization of human aortic VICs primary cultured in hypoxic conditions. Our previous study demonstrated that hypoxia is effective for the expansion of VICs isolated from both calcified and non-calcified tissues [13]. In the current study, to characterize hypoxic culture (hypo-VICs), we examined the gene expression profiles using RNA-sequencing and evaluated their ability to differentiate into osteoblasts. Our research purpose was to optimize VIC culture conditions for drug screening and functional analysis of drug target proteins before clinical application.

## 2. Results

### 2.1. Low Oxygen Tension Promotes VIC Proliferation

**Figure 1:**
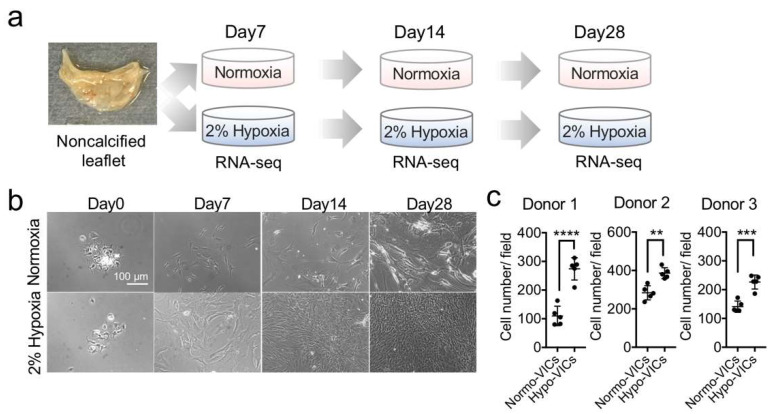
VICs exhibit increased growth under hypoxia. (**a**) Schematic of experimental strategy for RNA-sequencing. (**b**) Phase-contrast micrographs of VICs cultured under normoxia and 2% hypoxia at 0, 7, 14, and 28 d. Bar = 100 μm. (**c**) Quantifications of proliferation in hypo- and normo-VICs. ** *p* < 0.01, *** *p* < 0.001, **** *p* < 0.0001.

We isolated VICs from patients (three donors) without calcification who underwent aortic valve replacement for aortic valve regurgitation and cultured them for 3 weeks under 2% O_2_ (hypoxia) and normoxia (Figure 1a). While there was no difference in morphology at day 7 between the hypoxia and normoxia conditions, higher cell densities were observed under hypoxia than under normoxia (Figure 1b). This tendency continued for 28 d (Figure 1b). Cell counts confirmed this increase (Figure 1c). These phenotypes were observed in the VICs obtained from all three donors. These data indicate that hypoxic culture is suitable for maintaining primary VICs growth.

### 2.2. Gene Expression Profiling

**Figure 2:**
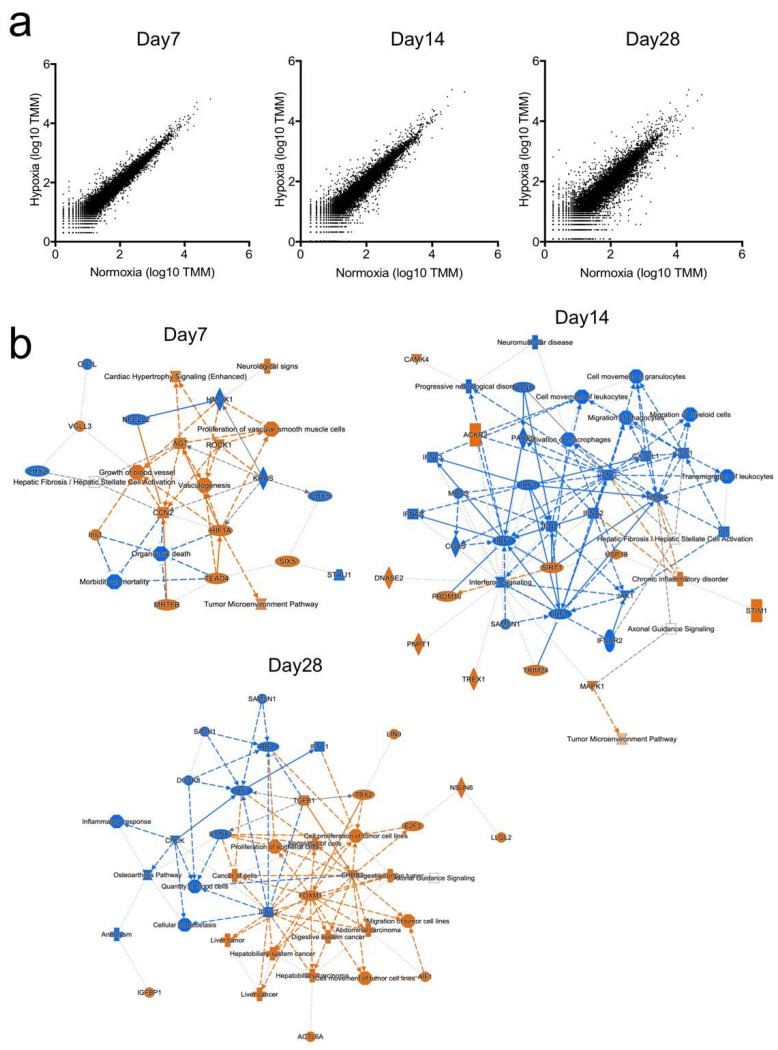
Gene expression in hypo- and normo-VICs. (**a**) Scatter plots of RNA-sequencing data (log_10_ TMM) at 7, 14, and 28 d. (**b**) Graphical summaries of IPA pathway analysis at 7, 14, and 28 d. Genes significantly upregulated in normo-VICs and hypo-VICs are denoted in blue and orange, respectively.

To explain the molecular-based differences in cell growth between hypo-VICs and normo-VICs, we performed RNA-sequencing. As shown in Figure 2a, scatter plots of gene expression levels in hypo-VICs and normo-VICs gradually expanded as a function of time, indicating that cell fates were clearly distinguished (Figure 2a). Ingenuity pathway analysis (IPA) showed that the expression of hypoxia inducible factor 1α (HIF1α)-dependent hypoxic-response genes, including those related to proliferation of vascular smooth muscle cells, vasculogenesis, and growth of blood vessels were strongly induced in hypo-VICs at day 7 (Figure 2b). In contrast, inflammation-related signaling pathways, such as interferon regulatory factors (IRFs), interferon-lambda (IFNL1), and activation/migration of macrophages, were strongly activated in normo-VICs at day 14 (Figure 2b). At day 28, cancer cell proliferation- and movement-related genes were upregulated in the hypo-VICs, while inflammation response-related genes were continuously elevated in normo-VICs (Figure 2b).

**Figure 3:**
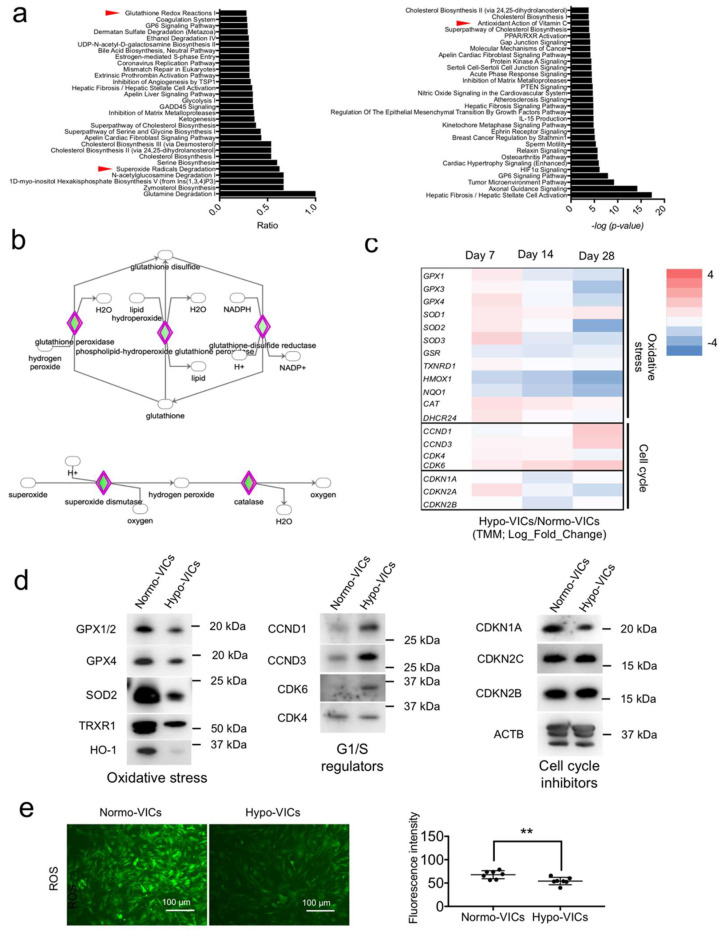
Expression of oxidative stress and cell-cycle regulators in normo- and hypo-VICs. (**a**) Top 30 candidates from canonical pathways (ratio and significance) from IPA analysis. Red arrowheads indicate canonical pathways pertaining to redox. (**b**) IPA pathway maps for glutathione redox reactions I and superoxide radical degradation. The rhombus colored in green denotes genes downregulated in hypo-VICs relative to normo-VICs. (**c**) Heatmap of expression of oxidative stress- and cell cycle-related genes from RNA-sequencing data (TMM). (**d**) Western blot detection of markers for oxidative stress and cell-cycle regulation. (**e**) Fluorescence micrographs of ROS. Bar = 100 μm (left panel). ROS quantification (right panel). ** *p* < 0.01.

To identify master regulators that could explain these differences in cell growth and morphology, gene enrichment analyses based on canonical pathways were conducted. Oxidative stress-marker genes categorized in glutathione redox reactions I and superoxide radical degradation were strongly enriched in expression (Figure 3a). The antioxidant category of Vitamin C-related genes was also significantly enriched in statistical analyses using Fisher’s exact test (Figure 3a). Signal maps illustrate the upregulation in expression of genes related to glutathione and superoxide-metabolic signaling in normo-VICs (Figure 3b). A heatmap of expression levels illustrates downregulation (in hypo-VICs relative to normo-VICs) in expression of glutathione peroxidase and superoxide dismutase family genes (Figure 3c, top). Additionally, upregulation of cell-cycle progression-related genes, such as cyclin (CCN) D1 and 3 and cyclin-dependent kinase (CDK) 4 and 6, and the downregulations of gene expressions related to cell cycle arrest, such as cyclin-dependent kinase inhibitor (CDKN) 1A, 2A, and 2B, was observed (Figure 3c, bottom). Protein expression was assessed using western blotting (Figure 3d), indicating that oxidative stress is strongly induced in normo-VICs, but not in hypo-VICs. Reactive oxygen species were increased in VICs cultured under normoxia (Figure 3e).

### 2.3. Differentiation of Hypo-VICs into Osteoblasts

**Figure 4:**
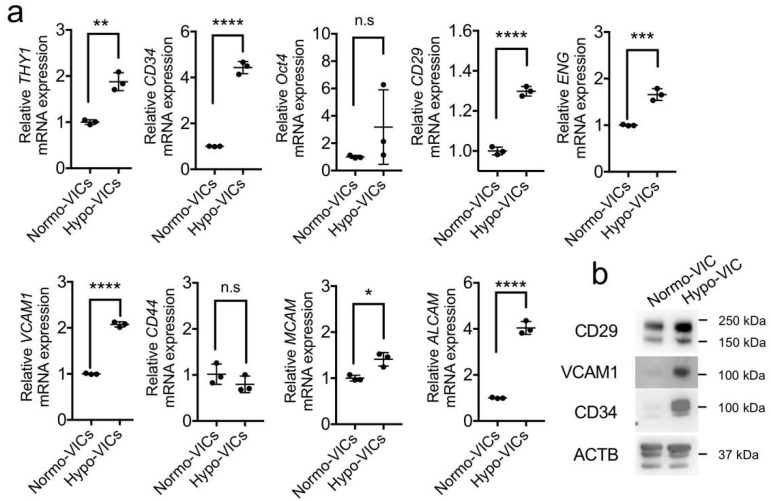
Expression of mesenchymal and hematopoietic stem cell markers in hypo- and normo-VICs. (**a**) Quantifications of mRNA levels for *THY1, CD34, Oct4, CD29, ENG, VCAM1, CD44, MCAM*, and *ALCAM* (*n* = 3). Expression levels were normalized to those of *GAPDH*. Data are presented as means ± SE. (**b**). Validation of CD29, VCAM1, and CD34 upregulation in hypo-VICs by western blotting. * *p* < 0.05, ** *p* < 0.01, *** *p* < 0.001, **** *p* < 0.0001. n.s.: Not significant.

To investigate the multipotentiality of hypo-VICs, expression levels of marker genes for MSCs and hematopoietic cells were quantified. The expression levels for *THY1/CD90, CD34, CD29, ENG, VCAM1, MCAM,* and *ALCAM* were significantly upregulated in the hypo-VICs than that in the normo-VICs, whereas levels of *CD44* and *OCT4* mRNA expression remained unchanged (Figure 4a). Increases in CD29, VCAM1, and CD34 protein expression were also observed (Figure 4b).

**Figure 5:**
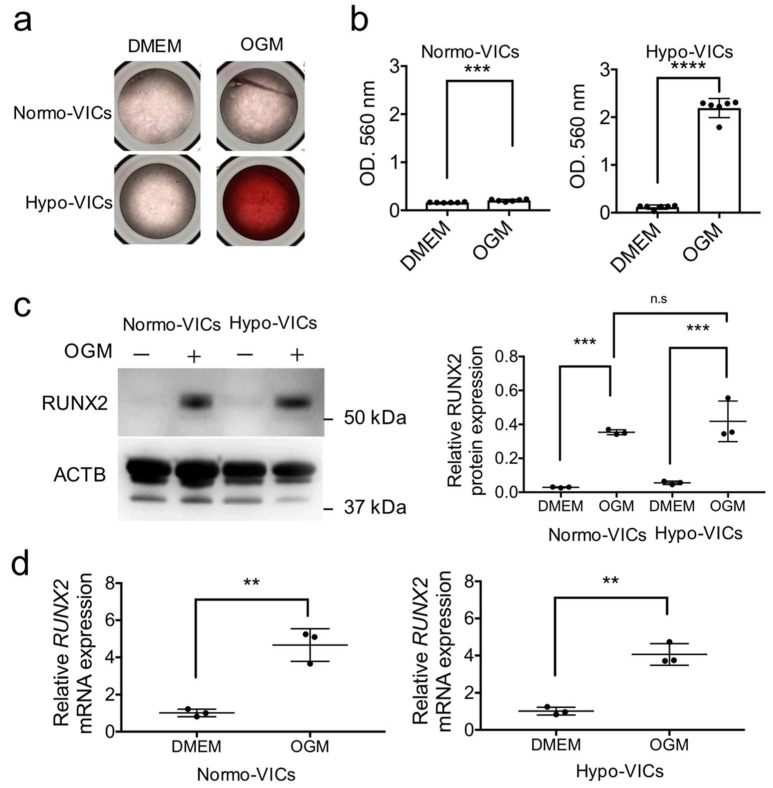
Differentiation of hypo- and normo-VICs into osteoblasts. (**a**) Photographs of DMEM- and osteogenic medium (OGM)-treated normo- and hypo-VICs stained by Alizarin red. (**b**) Calcium content quantified using cetylpyridinium chloride; absorbance was measured at 560 nm. Data are represented as means ± SE. (**c**) Western blot detection of RUNX2 in DMEM- and OGM-treated normo- and hypo-VICs (left panels). Beta-actin expression was used for normalization. RUNX2 protein expression in DMEM- and OGM-treated normo- and hypo-VICs (right panels). (**d**) Quantitative PCR data for RUNX2 mRNA expression in DMEM- and OGM-treated normo- and hypo-VICs. ** *p* < 0.01, *** *p* < 0.001, **** *p* < 0.0001. n.s.: Not significant.

To examine whether hypo-VICs are able to differentiate into osteoblasts, we cultured them in an osteoblast differentiation medium. As shown in Figure 5a, increased numbers of alizarin red-positive osteoblastic cells were observed in hypo-VICs. Quantification of calcium deposition confirmed significant elevations in calcification in hypoxic culture (over 18-fold) (Figure 5a); in contrast, normo-VICs were only slightly calcified after induction of osteogenesis (over 1.2-fold) (Figure 5b). The expression of RUNX2, a master regulator of osteoblast differentiation, was significantly increased by osteogenic differentiation induction in both normo- and hypo-VICs; however, there was a larger increase in hypo-VICs (Figure 5c). This increase also was observed by quantification of RUNX2 mRNA levels (Figure 5d).

### 2.4. Expression of the Oxidative Stress Marker SOD2 Is Increased in VICs from Calcified AS Tissues

**Figure 6:**
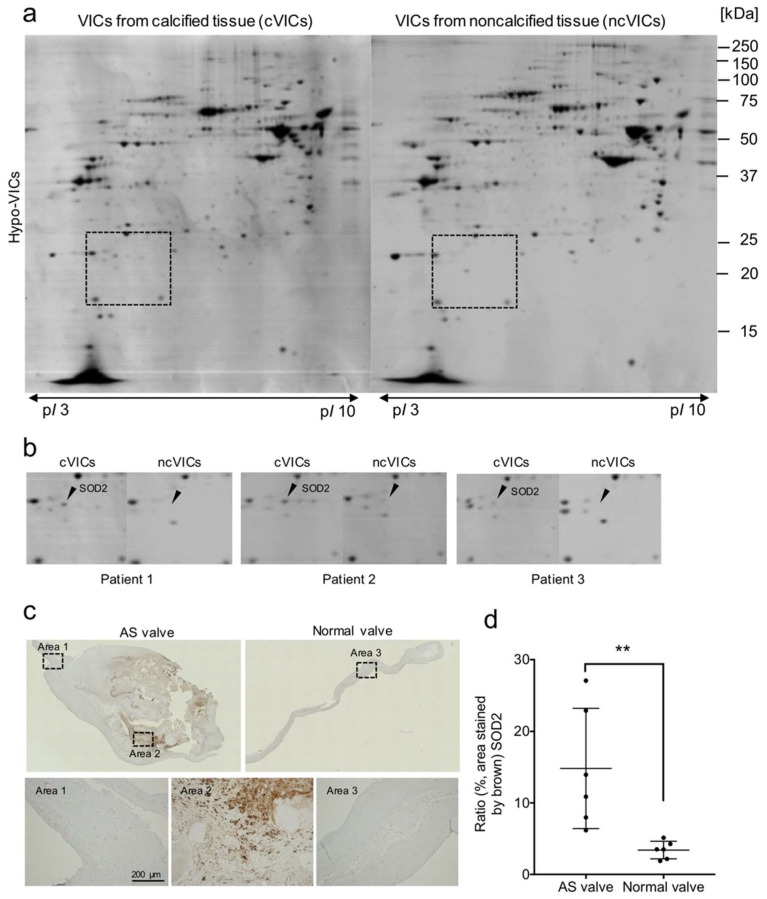
Expression of the oxidative stress marker SOD2 is increased in VICs from calcified AS tissues (cVICs). (**a**) 2DGE from lysates of cultured cVICs and ncVICs from patient 1. (**b**) Left panels, enlargements of the region surrounded by broken lines in (**a**) (patient 1). Middle and right panels show the same regions enlarged from 2DGE images of patients 2 and 3. (**c**) Immunohistochemical detection of SOD2 in AS and normal valves. Lower panels show enlarged views of areas 1–3 (boxes in upper panels). (**d**) Quantification of SOD2-stained areas; data are represented as means ± SE (*n* = 6). ** *p* < 0.01.

To determine whether hypo-VICs may have utility in molecular screening for therapeutics for the treatment of aortic stenosis (AS), we isolated VICs from calcified (cVICs) and non-calcified tissues (ncVICs) of AS valves and cultured them in hypoxic (2% O_2_) conditions, then performed two-dimensional gel electrophoresis. As shown in Figure 6a, we found a protein spot migrating at 21 kDa specific to cVICs (Figure 6a). This spot was detected in the cell lysates of cVICs from all three patients (Figure 6b). Nano liquid chromatography-mass spectrometry (LC-MS/MS) revealed that the 21 kDa-protein spot was SOD2, an oxidative stress marker (Table 1). Immunohistochemical staining showed that SOD2 was present in mesenchymal cells around calcified areas, but not around non-calcified areas. Furthermore, the expression of SOD2 in AS valves (*n* = 6) was significantly upregulated over that in normal valves (*n* = 6).

## 3. Discussion

We have provided the first molecular evidence demonstrating the benefits of hypoxic culture for VICs. Hypoxia promoted VIC proliferation, while normoxia inhibited growth and induced upregulation of the expression of oxidative stress-related genes. Several studies have demonstrated oxidative stress-induced cell senescence with growth arrest. Brandl et al. [14] reported that the growth of MSCs decreased with oxidative stress, identifying senescent morphologies with upregulation of CDKN1A expression [14]. Sun et al. reviewed the effects of exposing cells to oxidative stress, including senescence with proliferative arrest, CDKN1A upregulation, morphological changes, and senescence-associated, secretory phenotype related-cytokine production [15]. We found that normo-VICs exhibited dramatic morphological changes (Figure 1b), increased expression of CDKN1A and oxidative stress marker genes, and activation of the inflammation signal cascade, suggesting that normo-VICs are impaired in cell-cycle progression due to oxidative stress-induced cell senescence. In contrast, we also observed increased expression of cell-cycle progression-related genes in hypo-VICs. HIF1α-mediated signaling was also activated in the hypo-VICs. The expression levels of vascular endothelial growth factor (VEGF), which is a major target gene of HIF1A, and fibroblasts growth factor were also elevated in the hypo-VICs within 28 d. Previous studies have shown that proliferation of human adipose-derived stem cells under hypoxia was promoted via activation of the HIF1α-targeting VEGF and FGF signal pathways [16]. Hypoxia-induced HIF1α signaling also contributes to the suppression of MSC apoptosis [17]. The expression of CCND1 has been shown to be upregulated by the activation of HIF1α signaling [18]. Collectively, our data and previous studies indicate that the activation of HIF1-mediated signal transduction in hypo-VICs and oxidative stress-induced cell senescence in normo-VICs are likely explanations for the critical differences in proliferation observed between the two types of cells.

Our current study also showed changes in expression of markers of mesenchymal stem cell in the hypo-VICs. Thy1 is an MSC marker in multiple tissues [19]. The combined expression of Thy1 and CD29 has also been described in MSCs in human and mouse [20,21,22]. Additionally, ENG (CD105)- [23], VCAM-1 (CD106)- [24], MCAM(CD146)- [25], and ALCAM (CD166)- [5] positive cells were also reported as MSCs. These markers, but not CD44, which has been reported to be an MSC marker [26], were overexpressed in hypo-VICs relative to normo-VICs. Hypo-VICs predominantly differentiated to osteoblastic cells, suggesting multipotency. High CD34 expression was maintained in hypo-VICs, while the expression decreased in normo-VICs. While CD34-positive cells are well-defined as the hematopoietic stem cells in bone marrow, evidence for the expression of CD34 in multipotent adipose stromal cells has been published [27]. Yoshimura et al. demonstrated that CD34-positive cells contribute to osteoblast differentiation, although CD34 expression decreased as a function of time in culture [28]. Several researchers have reported that CD34-positive VICs exist in non-calcified, but not calcified, normal aortic valves [29,30]. Our data and previous studies suggest that high CD34 expression in hypo-VICs may be crucial for maintaining stemness, providing a source of osteoblast differentiation for calcification. Additionally, the features of hypo-VICs as a stem cell are also explained by both osteoblast differentiation assays but also by the gene expression. For example, keratin family genes, including *KRT10, KRT14, KRT16, KRT17, KRT18, KRT19, KRT7, KRT8, KRT80, and KRT86*, which are markers for epithelial cells, were strongly expressed in hypo-VICs but not in normo-VICs, suggesting that loss of mesenchymal identity in VICs following hypoxia may be crucial for obtaining stem-cell properties. Consistent with this, Mani et al. [31] has shown that epithelial-mesenchymal transitions generated stem-like cells [31]. In the future, lineage tracing of single cells will be essential to understand the roles of hypo-VICs during osteoblast differentiation.

Previous studies have shown that oxidative stress inhibits osteoblast differentiation. Bai et al. [32] demonstrated that oxidative stress activates extracellular signal-regulated kinase 1/2 (ERK1/2) and NF-κB signaling, which suppress osteoblast differentiation of bone cells [32]. Mody et al. [33] reported that hydrogen peroxide- or xanthine-mediated oxidative stress inhibits osteoblast differentiation of vascular and bone cells [33]. These results are consistent with our data, indicating that hypoxic culture has a large advantage in maintaining stemness and proliferation for investigations of the molecular mechanisms of VIC osteoblast differentiation. Furthermore, we identified SOD2 as a calcification-related protein in aortic VICs isolated from patients with AS. While an increase of SOD1 expression in AS patients was previously shown by proteomic analysis of whole-tissue lysates [34,35], evidence regarding tissue distribution and functional roles of SOD2 in valve calcification has not been reported.

A previous clinical study demonstrated different mechanisms of valve calcification between bicuspid patients and elderly patients in the context of VIC differentiation to osteocytes and chondrocytes [36]. These data also suggest that aberrant hemodynamic forces on bicuspid valves might promote osteoblast differentiation. *NOTCH1* mutations have been implicated in osteoblast differentiation via increasing BMP-2 expression [37]. Our osteoblast differentiation assay using hypo-VICs will be useful to address the functional roles of mutations affecting bicuspid valve function. Comparative investigations of tissue distributions and molecular roles of SOD2-positive VICs in bicuspid patients and elderly patients will be needed. 

Previous research has demonstrated that osteoblast-like cells are present in extensively calcified vascular vessels [38]. Mesenchymal stem cells, vascular smooth muscle cells, and endothelial progenitor cells have also been reported as progenitors of osteoblastic cells during vascular calcification [39]. We conclude that wider use of hypoxic culture methods will be applicable to the investigation of vascular calcification mechanisms and regenerative medicine concerning vascular and valvular diseases. 

The current study has several limitations. First, further evaluations of proliferation and expression of stem-cell markers in hypo-VICs derived from a larger number of patients with noncalcified valves will be essential for strengthening our conclusions. Second, increasing our understanding of differences in protein expression in hypo-VICs derived from calcified and noncalcified tissue using high-sensitivity mass spectrometry with more accurate quantifications should be conducted, given their potential for identification of novel therapeutic targets. 

## 4. Materials and Methods

### 4.1. Antibodies and Reagents 

Anti-glutathione peroxidase 1/2 (catalog no. sc-133160), anti-glutathione peroxidase 4 (catalog no. sc-166570), anti-superoxide dismutase 2 (catalog no. sc-137254), anti-TrxR1 (catalog no. sc-28321), and anti-heme oxygenase 1 (catalog no. sc-136960) antibodies were obtained from Santa Cruz Biotechnology (Santa Cruz, CA, USA). Anti-cyclinD1 (catalog no. 2978), anti-cyclinD3 (catalog no. 2936), anti-CDK6 (catalog no. 3136), anti-CDK4 (catalog no. 2906), anti-CDKN1A (catalog no. 2946), anti-CDKN2C (catalog no. 2896), anti-CDKN2B (catalog no. 4822), anti-beta1 integrin (CD29) (catalog no. 4706), and anti-RUNX2 (catalog no. 12556) antibodies were purchased from Cell Signaling Technology (Danvers, MA, USA). Anti-β-Actin antibody (catalog no. A5441) was obtained from Sigma-Aldrich (Saint Louis, MO, USA). Anti-VCAM1 antibody (catalog no. 66294-1-Ig) was obtained from Proteintech group (Rosemont, IL, USA). Anti-CD34 antibody (catalog no. 81289) was purchased from Abcam (Cambridge, MA, USA). Osteoblast-Inducer Reagent (catalog no. MK430) was obtained from Takara Bio. Inc. (Shiga, Japan).

### 4.2. Patients and Ethical Approval

The collection of human aortic valves was conducted under the approval of the Ehime University Internal Review Board (protocols 1509022 and 1603002). For evaluations of cellular characteristics, VICs were obtained from three donors (Supplemental Table S1) with aortic regurgitation (AR) who were underwent aortic valve replacements. For immunohistochemistry, aortic stenosis (AS) valve tissues were obtained from six patients who were diagnosed with severe AS and underwent aortic valve replacements (Supplemental Table S2). Six normal control valve tissues were obtained from autopsies (Supplemental Table S3). Before the procedure, written informed consent was obtained from all patients.

### 4.3. VICs Isolation and Culture

Isolation and culture of VICs from excised AR valves were performed as previously reported [13]. Briefly, valve leaflets were digested with collagenase (Worthington Biochemical, Freehold, NJ, USA) for 24 h, followed by plating onto culture dishes. VICs were maintained without passage in Dulbecco’s modified Eagle’s medium (DMEM) supplemented with 5% fetal bovine serum, penicillin, and streptomycin at 37 °C in an atmosphere containing 2% oxygen (hypo-VICs) or normal air (normo-VICs) for 7, 14, and 28 d. For two-dimensional gel electrophoresis, before incubation with collagenase, valves from patients with AS were categorized as calcified or non-calcified. Calcium content was quantified using Metallo Assays (Metallogenics Co., Ltd., Chiba, Japan).

### 4.4. VIC Proliferation 

VICs were isolated from collagenase-treated valve leaflets obtained from three donors with non-calcified aortic valves. After culturing for 28 d, they were fixed with 4% paraformaldehyde for 30 min. Nuclei were visualized using Hoechst 33258 and counted using Image Pro-Plus software (Media Cybernetics, Silver Spring, MD, USA).

### 4.5. In Vitro Osteoblast Differentiation

Normo-VICs and hypo-VICs were plated onto 96-well plates at 80% confluency, in DMEM or DMEM-based osteogenic medium (OGM) supplemented with Osteoblast-Inducer Reagent under normoxia for three weeks. Medium was changed every 7 d. Calcification of VICs was visualized using 1% Alizarin Red solution (Sigma-Aldrich) after fixation with methanol. Micrographs were captured using a BZ-X800 microscope (Keyence, Kyoto, Japan). Quantification was performed by measuring the absorbance (560 nm) of 100 nM of cetylpyridinium chloride (Wako, Japan) extracted from the stained VICs.

### 4.6. ROS Assays 

ROS accumulation was evaluated using the Highly Sensitive DCFH-DA-ROS assay kit (Dojindo Molecular Technologies, Kumamoto, Japan) according to the manufacturer’s instructions. Briefly, after washing hypo- and normo-VICs with Hanks’ Balanced Salt Solution (HBSS) twice, cells were treated with the highly sensitive DCFH-DA dye working solution for 30 min. Fluorescence signals were observed using a fluorescence microscope (Olympus, Tokyo, Japan). Quantification was performed using ImageJ software (NIH).

### 4.7. RNA Isolation and Sequencing

Total RNA was purified using ISOGEN II (Nippon Gene, Tokyo, Japan) according to the manufacturer’s protocol. RNA integrity of purified RNAs was measured using an Agilent 2100 Bioanalyzer (Agilent Technologies, Santa Clara, CA, USA). Library preparation and RNA-sequencing were performed as described [40]. Signal pathway analysis was conducted using QIAGEN Ingenuity Pathway Analysis (IPA) (Redwood City, CA, USA) using the trimmed mean of M values (TMM; range of expression-log-ratio, –1.0 down and 1.0 up).

### 4.8. Real-Time RT-qPCR

Five hundred micrograms of isolated total RNA were used for reverse-transcription using the High Capacity RNA-to-cDNA Master Mix (Applied Biosystems, Foster City, CA, USA) according to the manufacturer’s instructions. Quantitative real-time polymerase-chain reactions with the FastStart Universal SYBR Green Master ROX (Roche, Switzerland) were performed using the ABI 7500 Real-Time PCR system (Applied Biosystems). Primers are listed in Table 2.

### 4.9. Western Blotting

VICs were lysed using Laemmli sample buffer, followed by heating at 95 °C for 15 min. After measuring protein concentration using the RC DC Protein assay kit (Bio-Rad, Hercules, CA, USA), 20-µg total protein aliquots were subjected to 10–20% polyacrylamide gradient gel electrophoresis (Oriental Instruments, Tokyo, Japan). Proteins were transferred to a polyvinylidene fluoride (PVDF) membrane, blocked with Blocking One (Nakarai Tesque, Kyoto, Japan), and incubated with primary antibody overnight (1: 400 dilutions for all antibodies except to ACTB. 1:4000 dilutions for ACTB). After treatment with horseradish peroxidase-conjugated secondary antibody, chemiluminescence was detected using a LAS-4000 (GE Healthcare Life Sciences, South Logan, UT, USA). Band densities were quantified using ImageJ software.

### 4.10. Two-Dimensional Gel Electrophoresis (2D-GE) and LC-MS/MS

Lysis, extraction, and quantification of total proteins from ncVICs and cVICs were performed as previously described [41]. Two hundred-µg aliquots of total protein were subjected to 10–20% gradient gel electrophoresis (Oriental Instruments). Protein spots were visualized using the Bio-safe Coomassie blue gel staining reagent (Bio-Rad). The identification of a protein spot using nano LC-MS/MS was outsourced to Shimadzu Techno-Research, Inc (Kyoto, Japan).

### 4.11. Immunohistochemistry

Valve leaflets fixed with 4% paraformaldehyde were embedded in paraffin and sectioned at 5 µm. After deparaffinization, antigens were activated using citrate buffer (pH 6.0) by heating at 120 °C for 15 min. Anti-SOD2 antibody (1: 1000 dilution) was incubated with the sections overnight at 4 °C, followed by reaction with Histofine^®^ Simple Stain™ Mouse MAX PO peroxidase-conjugated anti-rabbit IgG (Nichirei Bioscience Inc., Tokyo, Japan). SOD2 was visualized using Histofine^®^ DAB substrate kits from Nichirei Bioscience Inc. (Tokyo, Japan). Stained areas were quantified using ImageJ software with the color deconvolution plug-in.

### 4.12. Statistical Analysis

All analyses were conducted using the GraphPad Prism software (GraphPad Prism Version 7.0c; GraphPad Software, San Diego, CA, USA). Statistical significance between two groups was calculated using Student’s *t*-test and the Mann–Whitney test for data with normality and non-normality, respectively. For more than three groups, statistical significance was evaluated using ANOVA, followed by Tukey’s multiple comparisons test.

## Figures and Tables

**Table 1:** Summary of LC-MS/MS analysis of 21 kDa-protein spot.

Accession No.	Gene Name	Score	Sequence Coverage (%)	Matched Peptides	Matched Amino Acids	Nominal Mass [kDa]	Calculated p*I*
P04179	*Superoxide dismutase [Mn], mitochondrial (SOD2)*	2922	68.92	15	222	24.73	8.25

**Table 2:** RT-qPCR primer sequences.

Target Gene	Direction	Sequence (5′-3′)
*Thy1*	forward	GAAGGTCCTCTACTTATCCGCC
	reverse	TGATGCCCTCACACTTGACCAG
*CD34*	forward	CCTCAGTGTCTACTGCTGGTCT
	reverse	GGAATAGCTCTGGTGGCTTGCA
*Oct4*	forward	CCTGAAGCAGAAGAGGATCACC
	reverse	AAAGCGGCAGATGGTCGTTTGG
*CD29*	forward	GGATTCTCCAGAAGGTGGTTTCG
	reverse	TGCCACCAAGTTTCCCATCTCC
*ENG*	forward	CGGTGGTCAATATCCTGTCGAG
	reverse	AGGAAGTGTGGGCTGAGGTAGA
*VCAM1*	forward	GATTCTGTGCCCACAGTAAGGC
	reverse	TGGTCACAGAGCCACCTTCTTG
*CD44*	forward	CCAGAAGGAACAGTGGTTTGGC
	reverse	ACTGTCCTCTGGGCTTGGTGTT
*MCAM*	forward	ATCGCTGCTGAGTGAACCACAG
	reverse	CTACTCTCTGCCTCACAGGTCA
*ALCAM*	forward	TCCAGAACACGATGAGGCAGAC
	reverse	GTAGACGACACCAGCAACAAGG
*RUNX2*	forward	TTCATCCCTCACTGAGAG
	reverse	TCAGCGTCAACACCATCA
*GAPDH*	forward	CATGAGAAGTATGACAACAGCCT
	reverse	AGTCCTTCCACGATACCAAAGT

## Data Availability

The raw data are available from the corresponding author upon reasonable request.

## Supplementary Materials

The following are available online at https://www.mdpi.com/article/10.3390/ijms221910534/s1.
