# Supplementary material for: Hypoxic Culture Maintains Cell Growth of the Primary Human Valve Interstitial Cells with Stemness
Source: Int J Mol Sci. 2021 Sep 29;22(19):10534. doi: 10.3390/ijms221910534 (PMC8508607; doi:10.3390/ijms221910534)
Supplement: Supplementary file 1 [file ijms-22-10534-s001.zip › ijms-1381633-supplementary.pdf]

SUPPLEMENTAL INFORMATION

Title: Hypoxic culture maintains cell growth of the primary human valve interstitial cells with stemness

Supplemental table 1

Characteristics of aortic regurgitation patients included in cell growth analysis

	Aortic regurgitation (n = 3)
Age, y	49 ± 22.2
Male, n (%)	3(100)
Body mass index	18 ± 2.0
Hypertension, n (%)	6 (100)
Smokers, n (%)	1 (33)
Dyslipidemia, n (%)	0 (0)
Diabetes, n (%)	0 (0)
Coronary artery disease, n (%)	0 (0)
Tricuspid aortic valves, n (%)	3 (100)

Data are given as n (%) or means ± standard deviations.

Supplemental table 2

Characteristics of aortic stenosis patients included in immunohistochemical analysis

	Aortic stenosis (n = 6)
Age, years	66 ± 7
Male, n (%)	5(83)
Body mass index	18 ± 3.2
Hypertension, n (%)	6 (100)
Smokers, n (%)	1 (17)
Dyslipidemia, n (%)	2 (33)
Diabetes, n (%)	1 (17)
Coronary artery disease, n (%)	1 (17)
Mean gradient (mmHg)	59 ± 16.8
Tricuspid aortic valves, n (%)	3 (50)

Data are given as n (%) or means ± standard deviations.

Supplemental table 3

Characteristics of autopsy patients included in immunohistochemical analysis

	Autopsy (n = 6)
Age, y	66 ± 13.3
Male, n (%)	2(33)
Hypertension, n (%)	3 (50)
Smokers, n (%)	1 (17)
Dyslipidemia, n (%)	0 (0)
Diabetes, n (%)	0 (0)
Coronary artery disease, n (%)	3 (50)
Time from death to autopsy (hrs)	59 ± 16.8
Tricuspid aortic valves, n (%)	6 (100)

Data are given as n (%) or means ± standard deviations.

Uncropped western blot image

Full-length blot for figure 3d

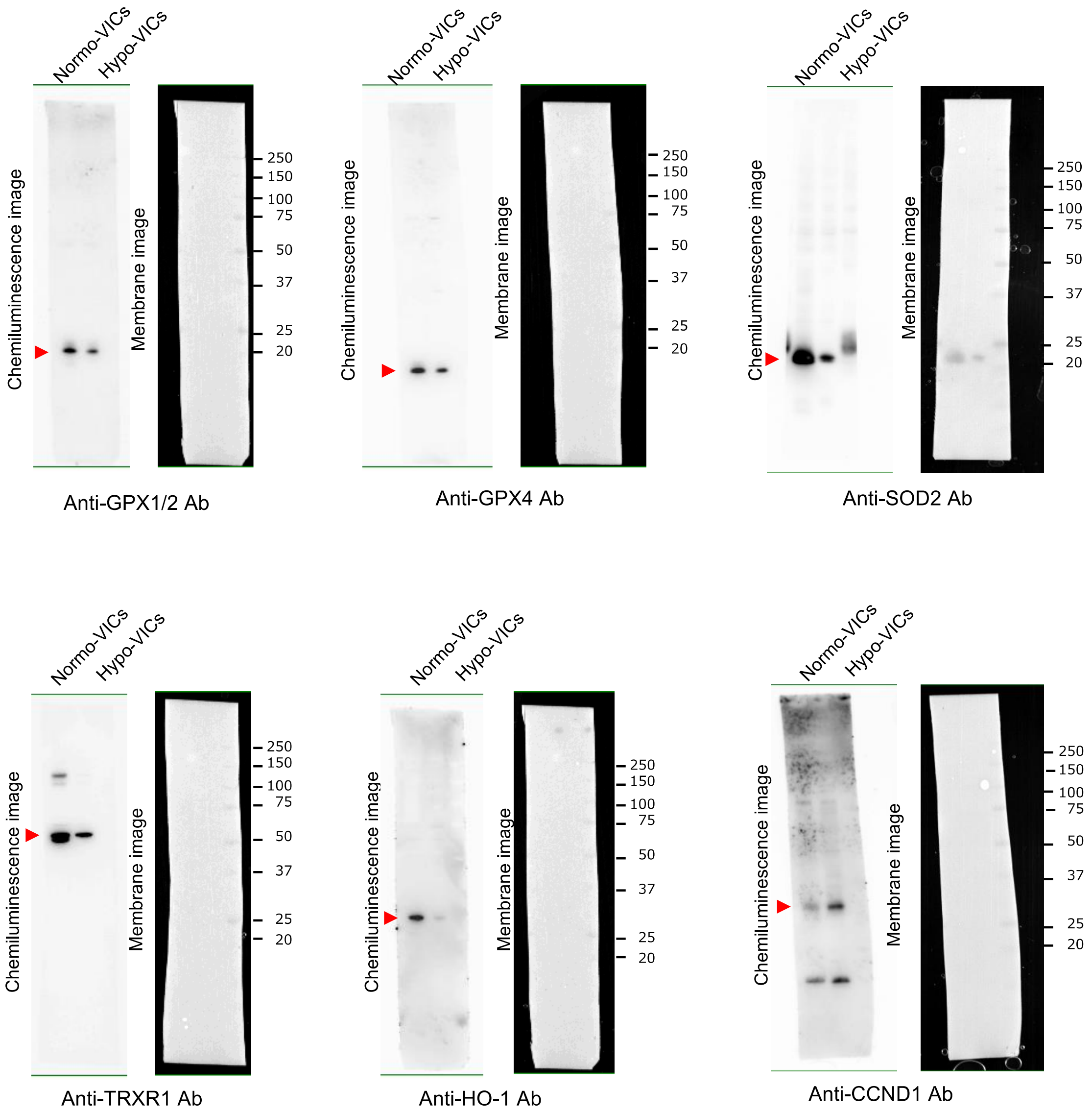

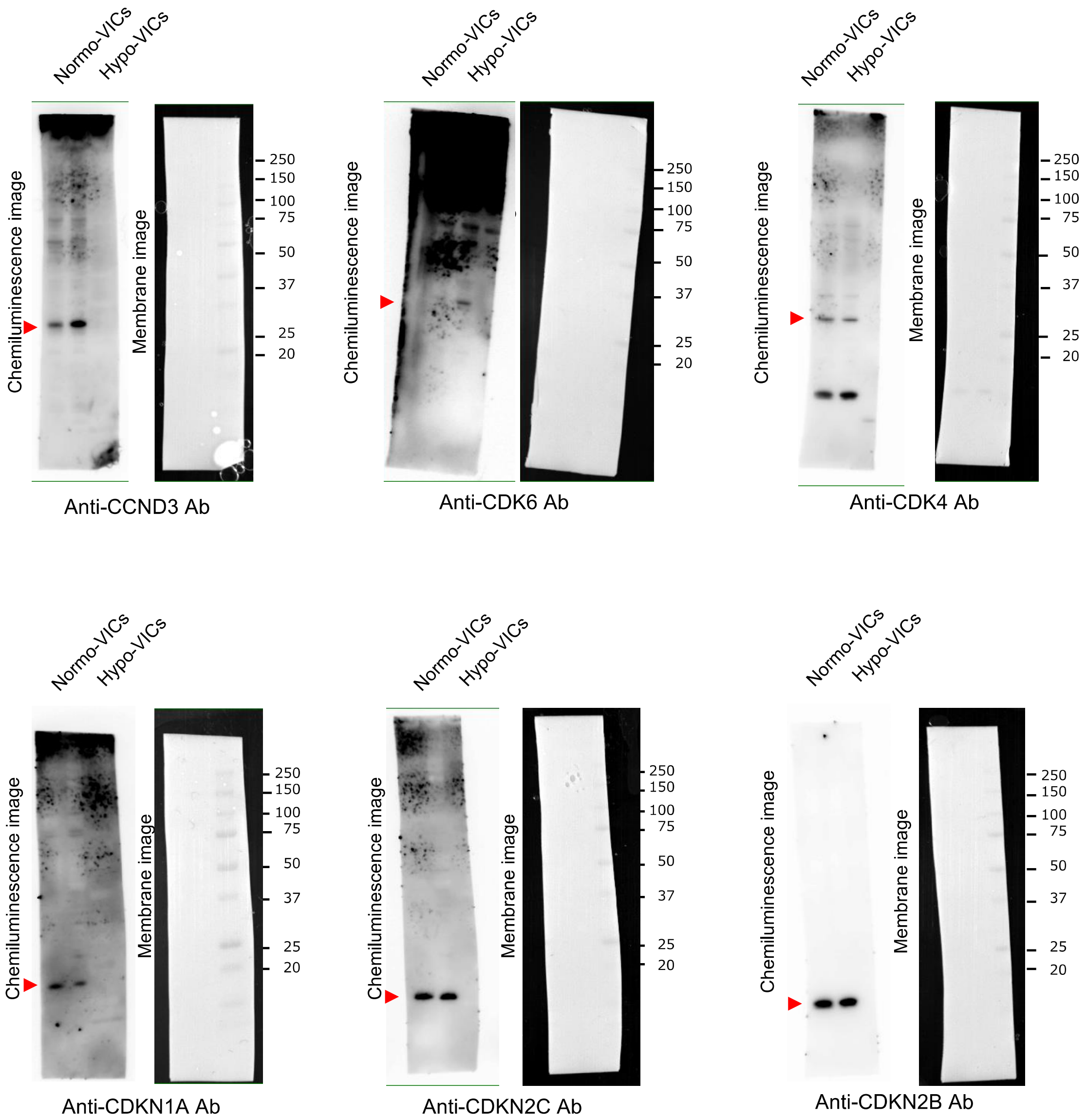
Normo-VICs
Hypo-VICs
Chemiluminescence image
Membrane image
250
150
100
75
50
37
25
20
Anti-CCND3 Ab
Anti-CDK6 Ab
Anti-CDK4 Ab
Anti-CDKN1A Ab
Anti-CDKN2C Ab
Anti-CDKN2B Ab

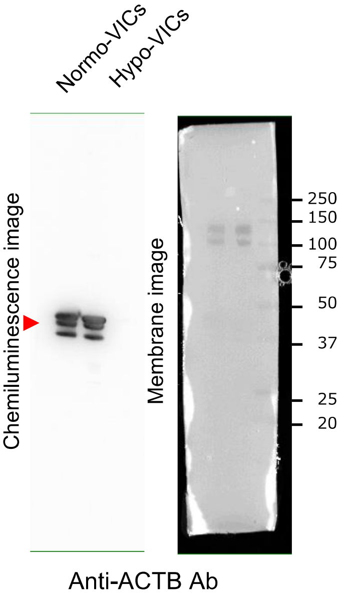
Normo-VICs
Hypo-VICs
Chemiluminescence image
Membrane image
250
150
100
75
50
37
25
20
Anti-ACTB Ab

Full-length blot for figure 4b

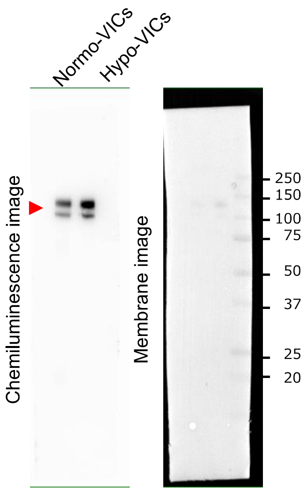

Anti-CD29 Ab

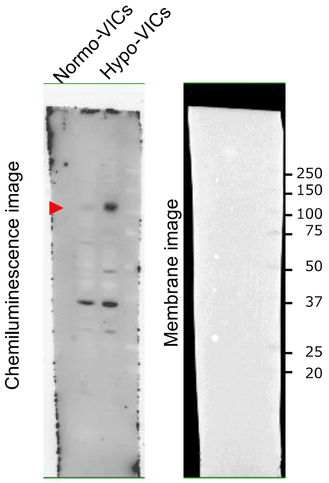

Anti-VCAM1 Ab

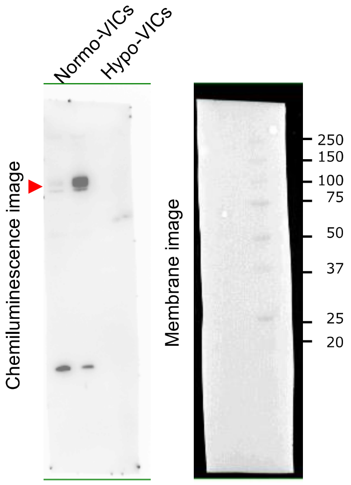

Anti-SOD2 Ab

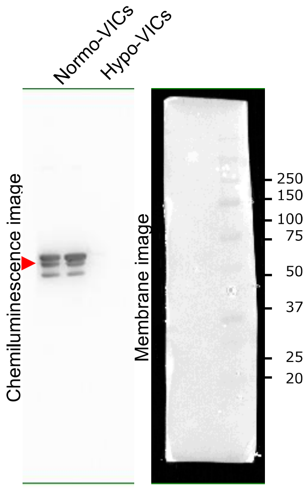

Anti-ACTB Ab

Full-length blot for figure 5c

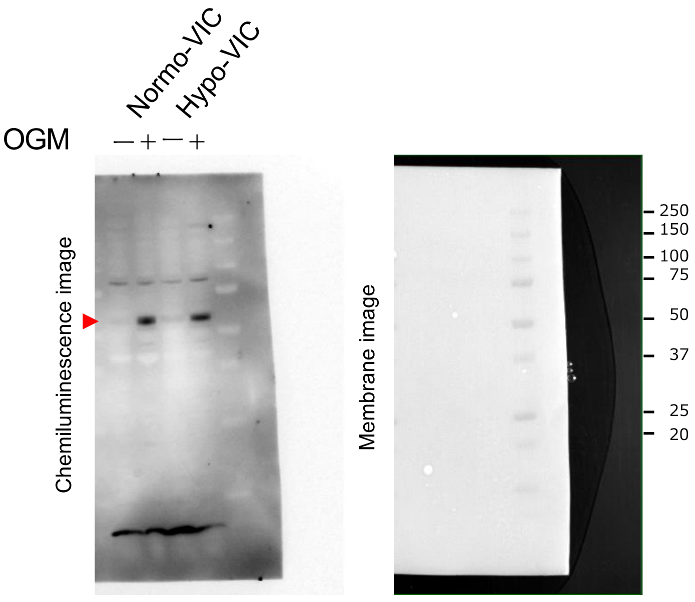

Anti-RUNX2 Ab

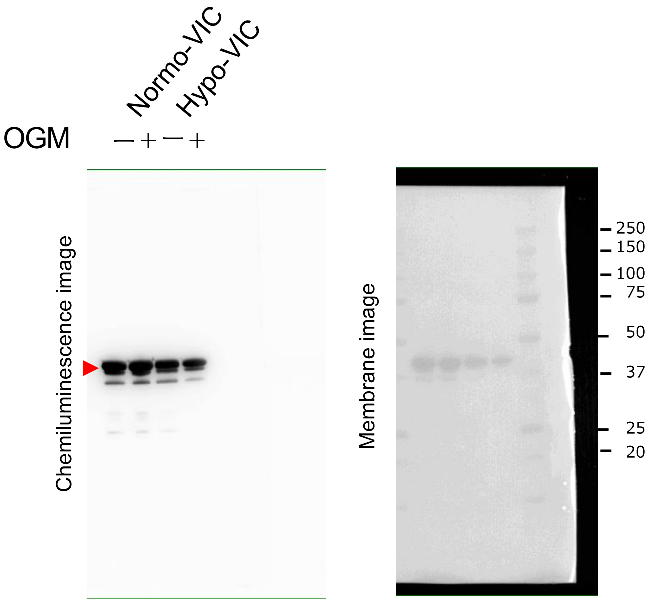

Anti-ACTB Ab
